# Clinical covariates influencing clinical outcomes in primary membranous nephropathy

**DOI:** 10.1186/s12882-023-03288-x

**Published:** 2023-08-10

**Authors:** Lukas Westermann, Felix A. Rottmann, Martin J. Hug, Dawid L. Staudacher, Rika Wobser, Frederic Arnold, Thomas Welte

**Affiliations:** 1 Department of Medicine IV, Medical Center, Faculty of Medicine, University of Freiburg, Freiburg, Germany; 2 Pharmacy, Medical Center, Faculty of Medicine, University of Freiburg, Freiburg, Germany; 3 Interdisciplinary Medical Intensive Care (IMIT), Medical Center, Faculty of Medicine, University of Freiburg, Freiburg, Germany; 4 Department of Cardiology and Angiology I, Heart Center, Faculty of Medicine, University of Freiburg, Freiburg, Germany; 5 Institute for Microbiology and Hygiene, Medical Center, Faculty of Medicine, University of Freiburg, Freiburg, Germany

**Keywords:** Primary membranous nephropathy, PLA2-R, THSD7A, Chronic kidney disease, Immunosuppression, Nephrotic syndrome, Rituximab

## Abstract

**Background:**

Primary membranous nephropathy (PMN) frequently causes nephrotic syndrome and declining kidney function. Disease progression is likely modulated by patient-specific and therapy-associated factors awaiting characterization. These cofactors may facilitate identification of risk groups and could result in more individualized therapy recommendations.

**Methods:**

In this single-center retrospective observational study, we analyze the effect of patient-specific and therapy-associated covariates on proteinuria, hypoalbuminemia, and estimated glomerular filtration rate (eGFR) in 74 patients diagnosed with antibody positive PMN and nephrotic-range proteinuria (urine-protein-creatinine-ratio [UPCR] ≥ 3.5 g/g), treated at the University of Freiburg Medical Center between January 2000 – November 2022. The primary endpoint was defined as time to proteinuria / serum-albumin response (UPCR ≤ 0.5 g/g or serum-albumin ≥ 3.5 g/dl), the secondary endpoint as time to permanent eGFR decline (≥ 40% relative to baseline).

**Results:**

The primary endpoint was reached after 167 days. The secondary endpoint was reached after 2413 days. Multivariate time-to-event analyses showed significantly faster proteinuria / serum-albumin response for higher serum-albumin levels (HR 2.7 [95% CI: 1.5 – 4.8]) and cyclophosphamide treatment (HR 3.6 [95% CI: 1.3 – 10.3]). eGFR decline was significantly faster in subjects with old age at baseline (HR 1.04 [95% CI: 1 – 1.1]).

**Conclusion:**

High serum-albumin levels, and treatment with cyclophosphamide are associated with faster proteinuria reduction and/or serum-albumin normalization. Old age constitutes a risk factor for eGFR decline in subjects with PMN.

**Supplementary Information:**

The online version contains supplementary material available at 10.1186/s12882-023-03288-x.

## Introduction

Membranous nephropathy (MN) is a rare kidney-specific autoimmune disease. The disease is caused by podocyte injury from immune complex deposits at the outer glomerular basal membrane (GBM). Histopathologically, this is characterized by thickening of the GBM. Typically, podocyte injury leads to impairment of the glomerular filter. Consequently, patients develop proteinuria, frequently in the nephrotic range [1–3]. The underlying pathophysiology defines two forms of MN: Primary membranous nephropathy (PMN) is caused by production of podocyte specific autoantibodies targeting the protein M-type phospholipase A2 receptor (PLA2R [4]), thrombospondin type-1 domain containing 7A protein (THSD7A [5]), neural epidermal growth factor-like 1 (NELL1 [6]), semaphorin 3b [7], and others (as reviewed in [8]). Secondary MN is caused by circulating immunoglobulins, produced during systemic diseases such as viral infections [9, 10] or certain malignancies [11, 12].

The course of PMN can be highly variable, ranging from spontaneous remission to progressive renal failure [13]. Approximately 34—40% of patients with severe proteinuria develop end stage renal disease (ESRD) [14, 15]. Hence, risk stratification is recommended to assess disease activity prior to therapeutic intervention [16]. Patients with low risk for progressive kidney injury (eGFR > 60 ml/min/1.73m^2^, proteinuria < 3.5 g/d, and serum albumin > 3 g/dl) often respond to antiproteinuric therapy with angiotensin-converting enzyme inhibitors (ACEI) or angiotensin receptor blockers (ARB). Cases with moderate to high risk qualify for immunosuppressive therapy with cyclophosphamide, rituximab, ciclosporin, corticosteroids, or a combination of those [16]. However, due to the rarity of the disease most studies incorporated comparatively small patient cohorts and thus evidence for immunosuppressive regimens is limited.

Effect sizes of both patient-specific and therapy-associated covariates in patients at risk for progressive kidney disease are poorly understood. This study aims to characterize the impact of covariates on disease activity (*i.e.*, proteinuria, hypoalbuminemia, and eGFR decline) in a cohort of patients with PMN.

## Methods

In this single-center retrospective observational study, we report data from the Renal division, University of Freiburg Medical Center, Germany. Inclusion criteria were age ≥ 18 years, biopsy-proven MN, detection of PMN-associated autoantibodies (PLA2R, or THSD7A in either patient serum or kidney biopsies), absence of secondary diseases associated with MN and nephrotic-range proteinuria (measured in spot urine samples by urine protein to creatinine ratio [UPCR] ≥ 3.5 g/g).

Subjects treated between January 1^st^, 2000 and November 30^th^, 2022 were screened for inclusion criteria. Age, sex, PLA2R-, THSD7A-status (other autoantibodies associated with MN were not tested in our department at the time of this study), serum creatinine levels, urine protein, urine creatinine, total cholesterol, HDL-, LDL-cholesterol, triglycerides, as well as time and dosage of treatment with ACEI/ARB and immunosuppressive agents (corticosteroids, rituximab, ciclosporin, cyclophosphamide) were extracted from electronic patient records. Patients were excluded at the timepoint of dialysis initiation or kidney transplantation. Estimated glomerular filtration rates (eGFR) were calculated using the CKD-EPI 2012 formula [17]. The follow-up time for each subject was defined as the time from inclusion to the last available laboratory measurement. Baseline characteristics were quantified during the first three months of follow-up.

The primary endpoint was defined as time to proteinuria/serum-albumin response (UPCR ≤ 0.5 g/g or serum-albumin ≥ 3.5 g/dl). The secondary endpoint was defined as time to permanent eGFR decline (time to decline of eGFR AND median of subsequent eGFR values relative to baseline ≥ 40%). Endpoints were individually analyzed using interval-censored time-to-event datasets. For interval censoring, right interval limits were the timespans between study inclusion and the endpoint-event. If the endpoint-event was not reached, the interval was right-unbounded. Left interval limits were the timespans between study inclusion and the last respective laboratory measurement prior to the endpoint-event. If no such measurement was available, the left interval limit was zero.

Statistical analysis and data visualization were performed using R 4.2.0 software [18], with the *survival* and *interval* packages [19, 20]. Multicollinearity was tested with the *performance* package [21]. If not stated otherwise, table data are presented as frequency and percentage, or median and interquartile ranges. For statistical comparison of categorical data, a Pearson’s chi square test was performed. For comparison of numeric variables, a Kruskal Wallis rank sum test was performed. Covariate-adjusted hazard ratios were estimated applying multivariate parametric regression models. Time-to-event curves were estimated using the Turnbull method on interval-censored datasets. To compare these time-to-event curves, *P*-values were calculated using log-rank like statistical tests. *P*-values < 0.05 were considered to be statistically significant.

## Results

### Baseline characteristics

We included *N* = 74 subjects in this study. The majority of subjects (67.6%) were male (Table 1). The median follow-up time was 31 (interquartile range [IQR]: 7—74) months. The median time since biopsy at study inclusion was 0 (IQR: 0—5) months. The median age at study initiation was 60 (IQR: 47—70) years. Subjects presented with moderately impaired renal function (median eGFR 55.5 [IQR: 30.5—84.1] ml/min/1.73 m^2^) and nephrotic-range proteinuria (median UPCR 6.0 [IQR: 4.2—9.3] g/g). Median serum-albumin levels were moderately decreased (2.9 [IQR:2.5 -3.3] g/dl). In line with MN treatment recommendations [16], most subjects (71.6%) were treated with ACEI or ARB, with *N* = 24 (32.4%) not receiving additional immunosuppression at baseline (Figure S1A). Notably, four subjects received treatment with both, ACEI and ARB at baseline, and in all instances, these treatments were administered sequentially. Immunosuppressive treatments included primarily prednisone (*N* = 22 [29.7%]), cyclophosphamide (*N* = 18 [24.3%]) or rituximab (*N* = 18 [24.3%]).

**Table 1:** Baseline characteristics

**Group**	**Covariate**	**Subjects (*N*)**	**Value**^a^
**General characteristics**	Female sex (*N*)	74	24.00 (32.43%)
	Age (years)	74	59.50 (46.50, 70.00)
	follow-up (months)	74	30.50 (7.25, 73.75)
	time since diagnosis (biopsy; months)	74	0.00 (0.00, 5.00)
**Renal parameters**	eGFR (ml/min/1.73m2)	74	55.52 (30.48, 84.09)
	UPCR (g/g)	74	5.97 (4.23, 9.27)
	Albumin (g/dl)	74	2.85 (2.50, 3.30)
**Lipids**	Cholesterol (mg/dl)	48	266.75 (230.75, 353.50)
	Triglycerides (mg/dl)	41	183.00 (138.00, 277.00)
	HDL-Cholesterol (mg/dl)	38	56.00 (46.25, 76.25)
	LDL-Cholesterol (mg/dl)	38	173.12 (132.00, 228.25)
**ACEI ARB treatment**	ACEI/ARB treatment (*N*)		53.00 (71.62%)
	ACEI equivalent (Ramipril [mg])	43	10.00 (5.00, 10.00)
	ARB equivalent (Candesartan [mg])	16	16.00 (16.00, 40.00)
**Immunosuppression**			
** Prednisone therapy**	Prednisone treatment (*N*)		22.00 (29.73%)
	Prednisone dose (mg)	22	20.00 (15.00, 34.38)
** Rituximab therapy**	Rituximab treatment (*N*)		18.00 (24.32%)
	Rituximab dose (mg)	18	1,000.00 (1,000.00, 1,000.00)
** Cyclophosphamide therapy**	Cyclophosphamide treatment (*N*)		18.00 (24.32%)
	Cyclophosphamide sum dose (mg)	18	2,900.00 (2,041.25, 4,063.75)
** MMF therapy**	MMF treatment (*N*)		2.00 (2.70%)
	MMF dose (mg)	2	1,500.00 (1,250.00, 1,750.00)

Doses for various administered ACEI and ARB were converted to ramipril or candesartan equivalence dose, respectively

*Abbreviations*: *ACEI* Angiotensin converting enzyme inhibitor, *ARB* Angiotensin-1 receptor blocker, *eGFR* Estimated glomerular filtration rate, *HDL* High-density lipoprotein, *LDL* Low-density lipoprotein, *MMF* Mycophenolate mofetil, *UPCR* Urine protein to creatinine ratio

^a^Discrete variables: Number, *N* (%); continuous variables: Median of each variable in the first three months of follow-up (IQR)

**Fig. 1:**
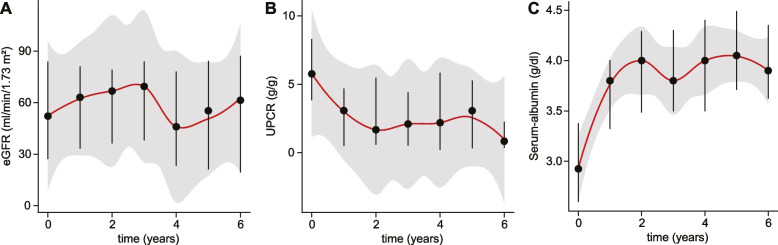
Change of outcome variables over time. Plots indicating median eGFR (**A**), UPCR (**B**), serum-albumin (**C**) levels per year in the study population in the first 6 years of follow-up. Error bars indicate 25% and 75% quartiles. A generalized additive regression model was fitted to the data (red lines). Grey shades indicate 95% confidence intervals of the regressions

All but two subjects receiving prednisone were either treated with cyclophosphamide (*N* = 15 [20.3%]), rituximab (*N* = 4 [5.4%]), or both (*N* = 1 [1.4%]; Figure S1A). Among the two subjects receiving monotherapy with corticosteroids, one had rituximab therapy planned but not yet initiated, while the other received monotherapy with steroids for an unrelated oncological condition. Of the two cases receiving MMF, one subject was treated with rituximab, the other had rituximab therapy planned but not yet initiated. Table 1 shows the properties of all analyzed covariates at baseline. eGFR, UPCR, serum-albumin levels and age were negatively correlated at baseline (Figure S1B). As expected, throughout the course of this study, UPCR and serum-albumin levels were inversely correlated (Fig. 1A-C) [22].

### Endpoint analysis

**Fig. 2:**
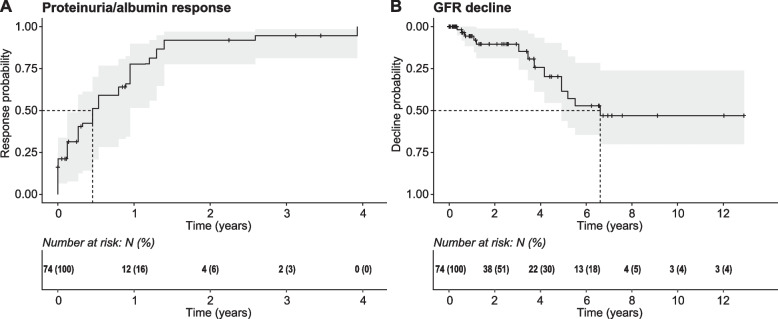
Univariate time-to-event analysis of primary and secondary outcome. **A**, **B** Time-to-event estimates fitted on interval-censored data for the primary endpoint (time to proteinuria/serum-albumin response; (**A**)) and the secondary endpoint (time to permanent eGFR decline (**B**)). Shading indicates 95% confidence intervals. Data are shown as global time to respective endpoints

Time-to-event analysis to assess the influence of disease-modifying covariates on all endpoints was performed on interval censored time-to-event data. The time to proteinuria/serum-albumin response (UPCR ≤ 0.5 g/g or serum-albumin ≥ 3.5 g/dl) was 167 days, with a probability of 78% (95% CI: 52—90%) to reach the endpoint after one year (Fig. 2A). The time to permanent eGFR decline (≥ 40% from baseline) was 2,413 days. The probability of reaching permanent eGFR decline after one year was 5.6% (95% CI: 0 – 12%; Fig. 2B).

Univariate time-to-event analyses showed significantly faster primary endpoint response for high vs low (> 3 g/dl vs ≤ 3 g/dl) serum-albumin levels and similar non-significant trends for low vs high (≤ 3.5 g/g vs ≥ 3.5 g/dl) UPCR levels at baseline (Figure S2A, B). eGFR stages at baseline did not significantly influence the time to proteinuria/serum-albumin response (Figure S2C). Among tested treatment covariates, treatment with cyclophosphamide, but neither treatment with ACE-inhibitors or ARB, nor treatment with prednisone or rituximab were associated with a significantly altered time to proteinuria/serum-albumin response (Figure S2D-G). Table S1 summarizes probabilities of proteinuria/serum-albumin response at 1 year for all covariates.

The time to permanent eGFR decline was not significantly influenced by serum-albumin or UPCR levels at baseline (Figure S3A, B). As expected, a trend towards faster eGFR decline in subjects presenting with low vs high eGFR at baseline could be observed (Figure S3C). Significance was not reached, as long-term follow-up data on subjects with low eGFR (< 30) was limited. Treatment with ACEI or ARB was associated with a non-significant trend for slower eGFR decline, while treatment with prednisone, cyclophosphamide or rituximab had no measurable effects on permanent eGFR decline in our study (Figure S3E-G). Table S1 summarizes probabilities of reaching eGFR decline at 1 year for all covariates.

**Fig. 3:**
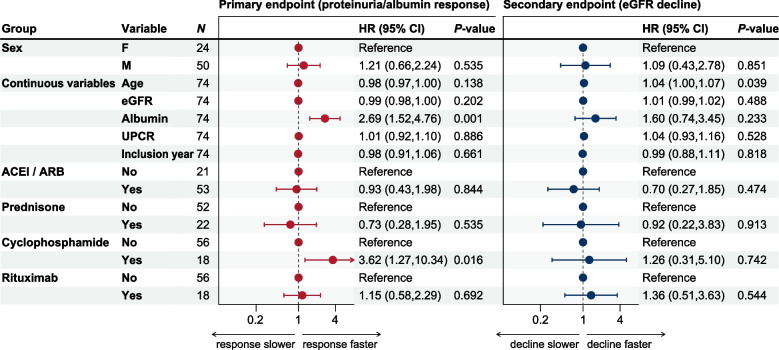
Multivariate endpoint analysis. Parametric regression models were fitted to interval-censored data. Endpoints: Primary endpoint (time to proteinuria/serum-albumin response; left panel), secondary endpoint (time to permanent eGFR decline; right panel). Abbreviations: eGFR, estimated glomerular filtration rate; inclusion year, year of inclusion in the study; UPCR, urine protein to creatinine ratio

We assessed the robustness of observed effects on the endpoints in multivariate parametric regression models (Fig. 3). High serum-albumin levels, but not low UPCR levels, were associated with faster proteinuria/serum-albumin response (HR 2.7 [95% CI: 1.5 – 4.8]). The year of treatment was not associated with altered proteinuria/ serum-albumin response or permanent eGFR decline. After covariate correction, treatment with cyclophosphamide was still associated with a significantly faster proteinuria/ serum-albumin response (HR 3.6 [95% CI: 1.3 – 10.3]), while treatment with prednisone or rituximab had no significant effects. A subtle, yet significant trend for faster eGFR decline was observed in subjects with old age (HR 1.04 [95% CI: 1 – 1.07]).

Multicollinearity analysis showed low correlation for all variables, endpoints, and regression models (Figure S4), corroborating independence of the variables and robustness of our results.

## Discussion

In this retrospective study we investigate the influence of clinical covariates on both, nephrotic symptoms, and renal function. Interestingly, serum-albumin, but not proteinuria levels at baseline correlate with faster recovery of nephrotic range proteinuria or hypoalbuminemia. This discrepancy may result from the applied methodology, as proteinuria estimation by UPCR inaccurately depicts high proteinuria levels (> 3 g/d) [23]. By contrast, serum-albumin levels are less prone to variation and may thus be more appropriate to assess proteinuria and guide therapy decisions. Of note, serum-albumin levels did not correlate with the eGFR response. This finding should be interpreted considering the moderate baseline hypoalbuminemia (2.9 g/l) observed in our cohort. Indeed, a previous study reported severe hypoalbuminemia (< 1.5 g/dl) to be associated with rapid eGFR decline [24]. Therefore, severity of hypoalbuminemia at presentation may be useful to identify patients at risk for both nephrotic symptoms and impaired renal function.

Regarding treatment-specific covariates, we did not observe significant effects of ACEI or ARB administration on nephrotic range proteinuria or hypoalbuminemia. This contrasts with antiproteinuric effects observed for ACEI treatment in nondiabetic nephropathies [25–27] and smaller studies reporting similar results in MN patients with low or moderate non-nephrotic proteinuria [28, 29]. Small sample sizes, short follow-up and a relatively low UPCR threshold (≤ 0.5 g/g) implemented in the definition of the primary endpoint might explain the lack of significant effects in our study.

Despite evolving therapeutic approaches for MN in recent years, there was no correlation between the covariate ‘inclusion year’ and time to reach both endpoints. This is likely attributed to a relatively recent inclusion of most subjects (median year at inclusion: 2016 [IQR: 2014—2019]).

Regarding immunosuppressive therapy, we observed significant antiproteinuric effects for cyclophosphamide, but not for prednisone or rituximab treatment. Low case counts in the respective subgroups of our study may explain the lack of significance, as previous studies have demonstrated antiproteinuric effects for both prednisone and rituximab [30–33]. It is worth noting that a recent investigation found a faster proteinuria response in membranous nephropathy with cyclophosphamide compared to rituximab-based treatment regimens [34]. Hence, the follow-up time of our study (median 30.5 months) might have been too short to observe significant effects for rituximab-based regimens. Similarly, inadequate follow-up time might as well explain why most covariates did not show significant effects for permanent eGFR loss.

This study has several limitations, the major being its retrospective character. Small sample size for subgroups treated with immunosuppressive agents, short follow-up time, and heterogeneous treatment regimens complicate the interpretation of outcomes. Furthermore, important covariates, such as histopathological data could not be assessed due to incomplete reporting.

Collectively, our study analyzes the influence of relevant clinical covariates on therapy response in patients with nephrotic-range proteinuria in PMN. We demonstrate that higher serum-albumin levels are a patient-specific predictor for faster recovery from nephrotic range proteinuria or hypoalbuminemia. As a therapy-associated covariate, treatment with cyclophosphamide results in faster recovery from nephrotic range proteinuria or hypoalbuminemia. Our findings encourage further research to quantify the influence of serum-albumin levels and other patient-specific predictors, as well as treatment-associated factors on PMN outcomes in greater detail. Prospective studies analyzing additional covariates are necessary to obtain a more comprehensive understanding of PMN outcomes.

### Supplementary Information

**Additional file 1. **

## Data Availability

The datasets analyzed in the current study are not publicly available to ensure privacy of research participants and comply with regulations of the ethics approval. Fully anonymized raw datasets analyzed in this study are available from the corresponding author on reasonable request. Computer codes are available on request from the corresponding authors (thomas.welte@uniklinik-freiburg.de; frederic.arnold@uniklinik-freiburg.de).

## Abbreviations

ACEI: Angiotensin converting enzyme inhibitor
ARB: Angiotensin receptor blocker
95% CI: 95% Confidence interval
CKD: Chronic kidney disease
eGFR: Estimated glomerular filtration rate
IQR: Interquartile range
TG: Triglycerides
HDL: High-density lipoprotein
LDL: Low-density lipoprotein
PMN: Primary membranous glomerulopathy
MMF: Mycophenolate mofetil
PLA2R: Phospholipase-A2 receptor
PRED: Prednisone
THSD7A: Against thrombospondin type-1 domain containing 7A protein
UPCR: Urine protein to creatinine ratio
RTX: Rituximab
