# Correction to: Clinical covariates influencing clinical outcomes in primary membranous nephropathy

**DOI:** 10.1186/s12882-023-03309-9

**Published:** 2023-09-03

**Authors:** Lukas Westermann, Felix A. Rottmann, Martin J. Hug, Dawid L. Staudacher, Rika Wobser, Frederic Arnold, Thomas Welte

**Affiliations:** 1 Department of Medicine IV, Medical Center, Faculty of Medicine, University of Freiburg, Freiburg, Germany; 2 Pharmacy, Medical Center, Faculty of Medicine, University of Freiburg, Freiburg, Germany; 3 Interdisciplinary Medical Intensive Care (IMIT), Medical Center, Faculty of Medicine, University of Freiburg, Freiburg, Germany; 4 Department of Cardiology and Angiology I, Heart Center, Faculty of Medicine, University of Freiburg, Freiburg, Germany; 5 Institute for Microbiology and Hygiene, Medical Center, Faculty of Medicine, University of Freiburg, Freiburg, Germany

Correction: BMC Nephrology (2023) 24:235 10.1186/s12882-023-03288-x

Following publication of the original article [1], we have been informed that author Thomas Welte was incorrectly affiliated.

The author affiliation has been updated above and original article has been corrected.
