# Supplementary material for: Clinical covariates influencing clinical outcomes in primary membranous nephropathy
Source: BMC Nephrol. 2023 Aug 10;24:235. doi: 10.1186/s12882-023-03288-x (PMC10413503; doi:10.1186/s12882-023-03288-x)
Supplement: Supplementary file 1 — Additional file 1. [file 12882_2023_3288_MOESM1_ESM.pdf]

Clinical covariates influencing clinical outcomes in primary membranous nephropathy

Lukas Westermann, Felix A. Rottmann, Martin J. Hug, Dawid L. Staudacher, Rika Wobser,

Frederic Arnold, Thomas Welte

Supplemental Material

Figures S1 – S4

Table S1

References to Supplementary Material

Supplemental Figures

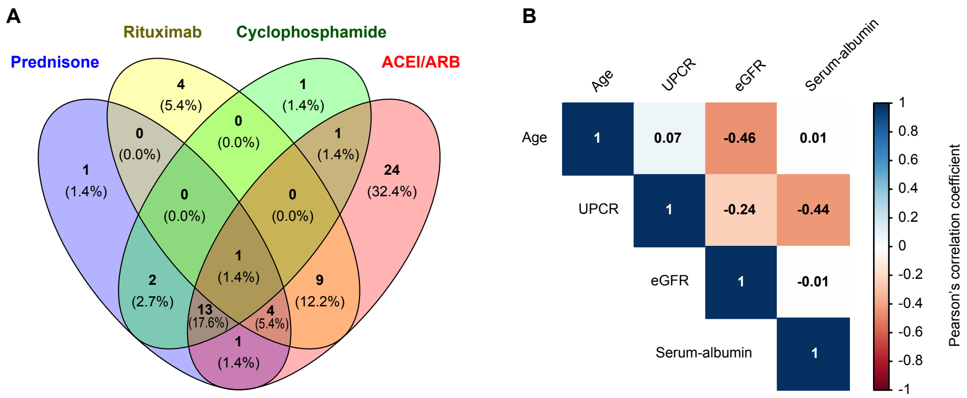

Figure S1. Baseline characteristics.
(A) Venn diagram showing the overlap of prednisone, rituximab, cyclophosphamide, and ACEI/ARB treatment. **(B)** Heatmap showing *Pearson's* correlation coefficients between selected variables at baseline. Data were aggregated by calculating the median for each subject and variable, ignoring NA values. Label legend: Age, age (years); Albumin, serum-albumin (g/dl); eGFR, estimated glomerular filtration range (ml/min/1.73m^2); UPCR, urine protein to creatinine ratio (g/g).

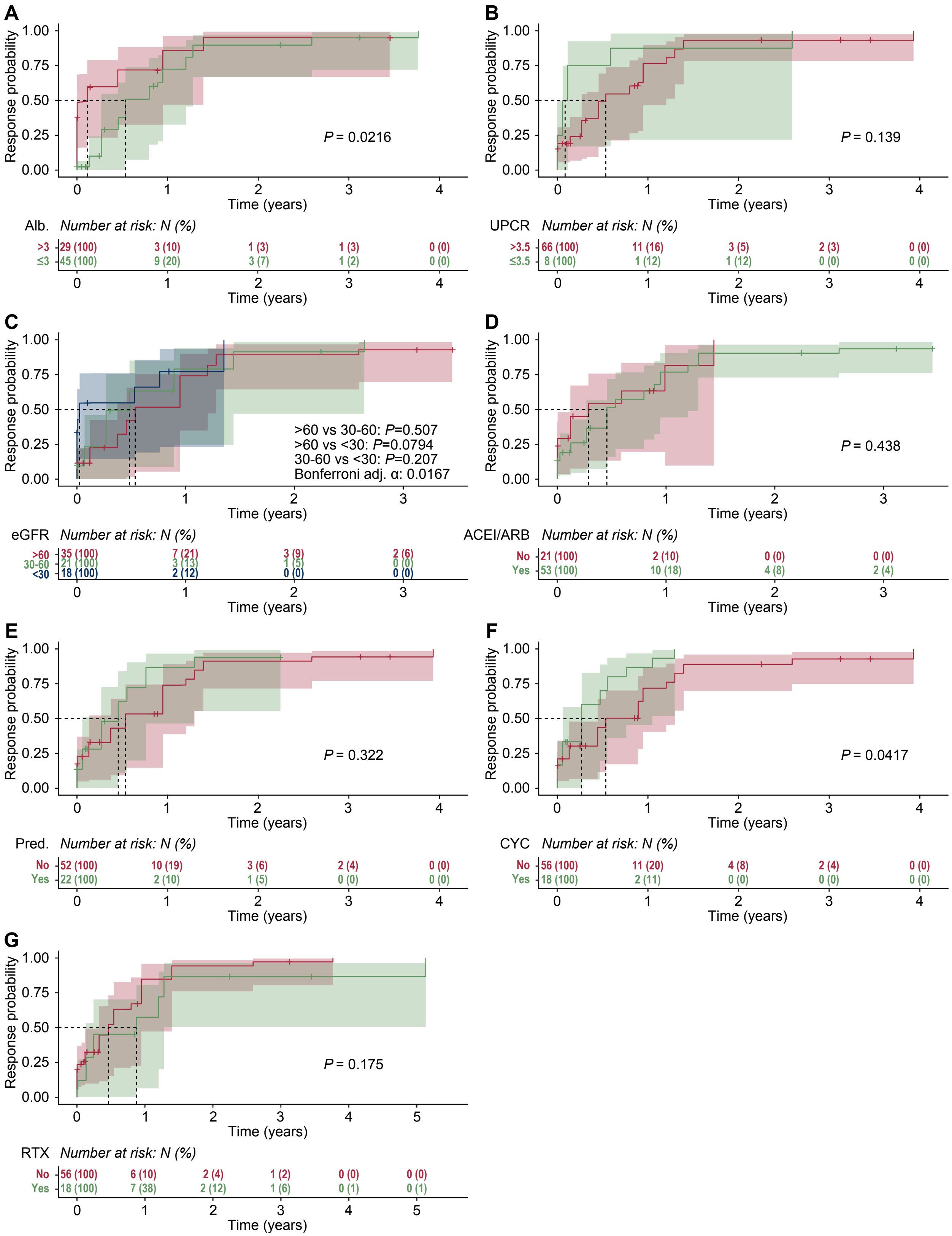

Figure S2. Univariate analysis primary endpoint (proteinuria/serum-albumin response).

(A-G) Time-to-event estimates fitted on interval-censored data for proteinuria/serum-albumin response. Stratification performed by serum albumin level (g/dl) at baseline **(A)**, UPCR level (g/g) at baseline **(B)**, eGFR (ml/min/1.73m^2) at baseline **(C)**, ACEI/ARB treatment at baseline **(D)**, prednisone treatment at baseline **(E)**, cyclophosphamide treatment at baseline **(F)**, and rituximab treatment at baseline **(G)**. Shading indicates 95% confidence intervals. Data are shown as global time to respective endpoints. *P* values calculated with log-rank like tests.

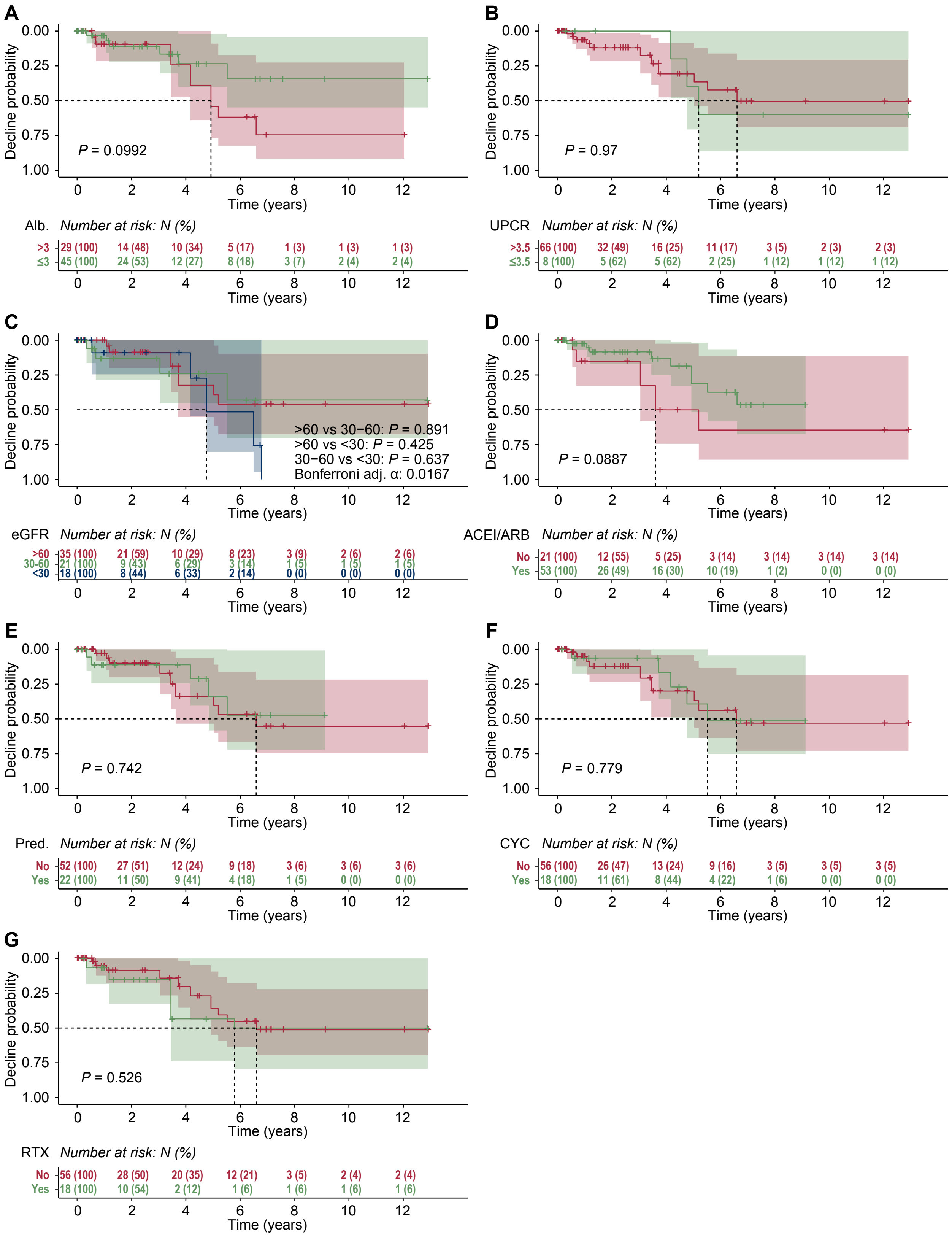

Figure S3. Univariate analysis secondary endpoint (permanent eGFR decline).
(A-G) Time-to-event estimates fitted on interval-censored data for permanent GFR decline (≥ 40% relative to baseline). Stratification performed by serum albumin level (g/dl) at baseline **(A)**, UPCR (g/g) level at baseline **(B)**, eGFR stage (ml/min/1.73m^2) at baseline **(C)**, ACEI/ARB treatment at baseline **(D)**, prednisone treatment at baseline **(E)**, cyclophosphamide treatment at baseline **(F)**, and rituximab treatment at baseline **(G)**. Shading indicates 95% confidence intervals. Data are shown as global time to respective endpoints. *P* values calculated with log-rank like tests.

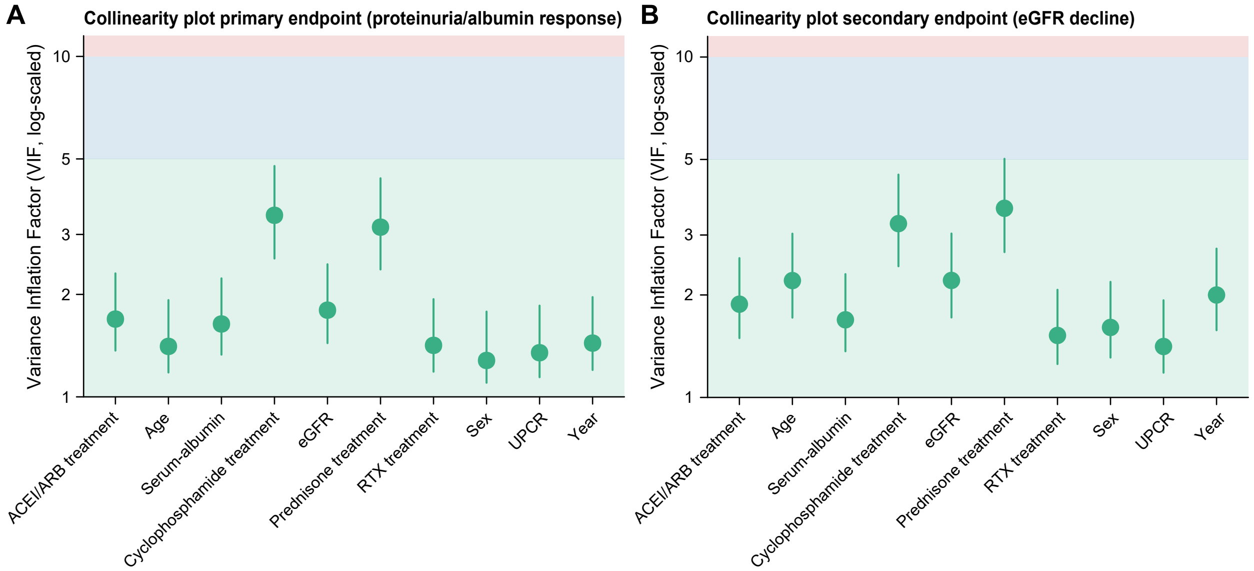

Figure S4. Multicollinearity analysis of multivariate models on interval censored data, showing variance inflation factors (VIF), calculated for the primary **(A)** and secondary **(B)** endpoints [1]. Bars indicate 95% confidence interval. Threshold for low correlarity: VIF < 5 [2].

Supplemental Table

Covariate	One-year probability (95% CI) of primary endpoint	One-year probability (95% CI) of secondary endpoint
**Serum albumin (g/dl)**		
>3	86% (47%, 96%)	9.6% (0%, 22%)
≤ 3	72% (33%, 89%)	3.1% (0%, 8.9%)
**UPCR (g/g)**		
> 3.5	77% (46%, 90%)	6.4% (0%, 13%)
≤ 3.5	88% (22%, 98%)	0% (0%, 0%)
**eGFR (ml/min/1.73m^2)**		
> 60	74% (35%, 90%)	0% (0%, 0%)
30-60	79% (25%, 94%)	13% (0%, 29%)
< 30	77% (23%, 93%)	9.1% (0%, 25%)
**ACEI/ARB treatment**		
No	82% (9.7%, 96%)	15% (0%, 33%)
Yes	77% (44%, 90%)	2.4% (0%, 6.9%)
**Prednisone treatment**		
No	74% (39%, 89%)	3.0% (0%, 8.6%)
Yes	87% (46%, 97%)	11% (0%, 25%)
**Cyclophophamide treatment**		
No	72% (40%, 87%)	5.3% (0%, 12%)
Yes	87% (45%, 97%)	6.3% (0%, 17%)
**Rituximab treatment**		
No	85% (47%, 96%)	5.4% (0%, 12%)
Yes	57% (6.4%, 81%)	6.7% (0%, 18%)

Table S1. Related to Figures S2 and S3.
Proteinuria/serum-albumin response (primary endpoint) and eGFR decline (secondary endpoint) probability at year one, stratified by various covariates. 95% confidence intervals in brackets.

References to Supplemental Material

1. Marcoulides KM, Raykov T: **Evaluation of Variance Inflation Factors in Regression Models Using Latent Variable Modeling Methods**. *Educ Psychol Meas* 2019, **79**(5):874-882.
2. James G, Witten D, Hastie T, Tibshirani R: **An introduction to statistical learning**, vol. 112: Springer; 2013.
